# Healthcare worker trauma and related mental health outcomes during the COVID-19 outbreak in New York City

**DOI:** 10.1371/journal.pone.0267315

**Published:** 2022-04-29

**Authors:** Bo Yu, Donell Barnett, Vidya Menon, Lara Rabiee, Yinelka Silverio De Castro, Moiz Kasubhai, Eren Watkins

**Affiliations:** 1 Department of Medicine, New York City Health + Hospitals, Lincoln Medical Center, Bronx, New York, New York, United States of America; 2 US Army Urban Augmentation Military Task Force, Bronx, New York, New York, United States of America; 3 Department of Ambulatory Care, New York City Health + Hospitals, Lincoln Medical Center, Bronx, New York, New York, United States of America; 4 US Army Public Health Center, Behavioral and Social Health Outcomes Program, Edgewood, Maryland, United States of America; University of Auckland, NEW ZEALAND

## Abstract

Healthcare workers (HCWs) faced a range of stressors during the coronavirus (COVID-19) pandemic, contributing to psychological stress. We use a psychological trauma framework to characterize the mental health burden for clinical and non-clinical healthcare worker occupations during the COVID-19 pandemic. The objective was to measure and characterize risk factors for trauma and anxiety-related mental health problems among HCWs at a public hospital in the epicenter of the COVID-19 pandemic in New York City (NYC). This study reports findings from a cross-sectional survey of NYC HCWs shortly after the initial 2020 infection surge. Over 800 hospital employees completed the survey that assessed professional quality of life indicators (compassion satisfaction [CS], burnout [BO], secondary traumatic stress [STS]), Coronavirus Anxiety (CS), Obsession with Coronavirus (OC), and PTSD symptoms. The survey also assessed pandemic-related work and life circumstances such as “do you have a family member or friend who tested positive for COVID”. Relatively small percentages of HCWs endorsed probable Coronavirus Anxiety (6%), PTSD (13%), and Coronavirus Obsession (21%). We observed higher proportions of Burnout (29%), Moderate or High Secondary Traumatic Stress (45%), and High Compassion Satisfaction (52%). Adjusted regression models showed important implications for prior behavioral/emotional health concerns among HCWs, providing care for a patient that died from COVID-19, and other characteristics. This study supports prior studies documenting the mental health consequences for the healthcare workforce during the COVID-19 pandemic. This study builds on that base by including non-clinical staff in the sample and assessing pandemic life-stressors such as caring for sick family members.

## Introduction

When considering the death toll, economic impact, and disruption to basic life routines like education, healthcare, and relationships, the novel Coronavirus COVID-19 ranks among the most serious in history. The COVID-19 pandemic prompted an aggressive public health response to include closing schools, businesses, and other establishments, all of which contributed to a rise in fear and anxiety among United States citizens [1]. A growing body of evidence documents to toll on healthcare workers (HCWs), who are particularly at risk for adverse mental health consequences given their proximity to those most severely impacted by the disease [2–5].

Empirical studies suggest that HCWs are at a disproportionate risk for adverse mental health consequences based on occupational, individual, and organizational factors [6]. Occupationally, researchers have reported that "frontline" workers [7], nurses in particular, are most at risk [6,8]. Some individual-level risk factors include pre-existing mental health concerns [9], younger HCWs [2], and women [10] carry more than expected COVID-19 mental health burden. While organizational-level factors are not as well-studied, news reporting suggests that scarce protective and treatment equipment and feelings of not being protected by employers are important organizational sources of stress [11]. These studies support the conclusion that COVID-19 pandemic has had significant adverse mental health consequences for healthcare workers. There are, however, important limitations to consider. First, the research is intricately linked to the chronological progression of the pandemic and the cultural context in which the study was conducted. Second, there is a variation in the outcomes of interest and inclusivity of the samples. Last, there is no apparent guiding theoretical framework to conceptualize the body of literature. For these reasons, crafting effective interventions is a more difficult task.

In March 2020, Jianbo [8] and colleagues offered the first report based on a sample of healthcare workers in Wuhan, China. Subsequently, researchers from Italy [12], India [3], Turkey [5], Spain [13], and other locations documented similar reports in addition to some multi-location studies [2,4,5]. This chronological progression of studies is especially relevant because having information is critical in an anxiety and trauma context. As the pandemic spread, later-affected countries had more time to learn about the disease, which likely meant precious additional time to prepare. Preparation and anticipation are critical buffers of psychological trauma because feeling prepared reduces the sense of being overwhelmed [14]. This progression also loosely follows a geographical and cultural path from China, to other countries in Asia, to Europe, and then to countries in the west. While countries in the west might have benefited from lessons learned in early-affected countries, like more global symptoms of COVID illness and useful public health prevention strategies, mental health symptoms are too closely bound by culture to base interventions off those early observations.

Methodological variation is the second concern. While previous studies used a wide variety of outcomes, there tended to be a narrow range of occupations included in HCW samples. Outcomes of interest range from formal diagnoses of depression, PTSD, anxiety, and insomnia, to other psychological constructs such as compassion fatigue and burnout, as well as more global wellness outcomes such as quality of life [7,8,10,12,15]. This broad range in outcomes of interest is important because it demonstrates the multi-faceted impact of working in healthcare during the COVID-19 pandemic. This variation, however, is not practical for practitioners aiming to craft interventions that remedy these adverse effects. Alternatively, there is notable prioritization of clinical staff in previous studies with a focus on physicians and nurses only. This focus carries with it some constraints. Clinical staff likely have some practice effects given that high intensity/high stress may be more routine for these roles when compared to the broader hospital-based healthcare workforce. Additionally, COVID-19 has been a challenge for the entire clinical and public health system. Still, we could find very few studies that sampled the broader class of the healthcare workforce, such as security, maintenance, and other allied specialties, all of whom made the COVID-19 response possible.

Both the range of outcomes studied in previous literature and the general exclusive focus on clinical staff may be tied to a lack of a broader theoretical framework guiding this line of study. While depression, anxiety, sleep problems, and global-wellbeing are expected mental health consequences, in the COVID-19 context, these responses were preceded by working in a high-stress traumatic environment. Therefore, we propose the stress-anxiety-trauma continuum as the best theoretical fit for understanding the HCW mental health burden. Moreover, this framework is consistent with clinical criteria for trauma and stress-related disorders [16].

Uncertainty is a key ingredient to stress and trauma. As the epidemic grew to be a pandemic, areas of the world affected later in the disease spread timeline had more information than those in the initial waves. It follows that the adverse symptoms would look different across studies in this area due to sample timing. Additionally, there is very little research on non-clinical healthcare workers such as security, administration, maintenance, and others critical to the success of the healthcare system. During COVID-19, the significant precautions and screening of patients and non-patients, handling the extraordinary number of corpses, and keeping records of patients and the deceased are primarily non-clinical roles. Those pandemic-related duties likely contributed to stress and trauma for non-clinical HCWs who might have been least familiar with handling that level of grief and loss. Additionally, in more urban areas of the United States, those occupational roles are likely to be held by racial and ethnic minority populations, representing a different set of risk and protective factors to consider when crafting interventions or support services.

The purpose of this study was to assess the extent of trauma-related mental health impacts among HCWs. Consistent with Carmassi and colleagues [17] guidance based on their study of HCW mental health following previous epidemics, this study also differs from earlier reports in three ways: 1) we considered protective factors in addition to risk factors, 2) we assessed trauma in both clinical and non-clinical staff, and 3) we evaluated non-work-related stressors such as being impacted by family and friends who have been infected with COVID-19 or are at risk for infection.

## Methods

After obtaining IRB approval (NYC Health and Hospitals, IRB #20–017), we conducted a cross-sectional anonymous web-based survey of HCWs at a Level 1 Trauma Care Center located in the Bronx, NYC burrough. We administered the survey in July 2020, four months after the city’s March COVID-19 surge. From March to December 2020, this site admitted approximately 1,200 COVID-19 patients, with more than half being severely or critically ill. We invited all hospital employees (n = 4,000) to participate in the survey via their work-issued email addresses. Overall, 1,113 (27%) initiated the online survey, and 889 (22% of the hospital staff) were included in the analytic population. The first page participants encountered included approved informed consent information. Participants who indicated “Yes, I agree and consent to participate in this study” were then presented with the remaining survey protocol. Those who indicated “No, I do not agree or consent” were directed to a close-out page containing useful resources and other support-related information for support. For inclusion in the analytic population, participants had to have a response for the occupation variable.

The survey included demographic questions such as age, sex (male, female or other), race (Asian, Black, multiple or other races, and White), ethnicity (Latinx, Non-Latinx), and education level (high school degree or lower, some college but no degree, Associate degree, Bachelor degree, Graduate degree). We also included occupation-related questions such as role in the hospital (e.g. physician, nurse, allied healthcare worker, hospital administration and operations support) and length of time in career, and health characteristics such as whether the respondent had a behavioral or emotional health concern before the pandemic.

We chose a trauma-oriented theoretical framework to assess healthcare worker mental health. Our outcomes of interest were symptoms of post-traumatic stress disorder (PTSD), burnout (BO), secondary traumatic stress (STS), compassion satisfaction (CS), Coronavirus Anxiety (CA), and Obsession with Coronavirus (OCS). We used The Primary Care-PTSD 5 [18] screener to assess past month PTSD symptoms. The PTSD 5 is a validated five-item measure to detect probable disorder based on the Diagnostic Statistical Manual of Mental Disorders (DSM-5) criteria. Respondents endorse a "yes" or "no" response to five questions with one point for each "yes" response. We used a cut-point score of "3" to detect probable PTSD.

To assess trauma response associated with working in a healthcare capacity, we used the Professional Quality of Life scale (ProQoL) [19]. The ProQoL is comprised of 30-items whereby respondents rate each item using a five-point likert with "1" being "Never" and "5" being "Very Often". The ProQoL contains the three subscales Compassion Satisfaction (CS), Burnout (BO), and Secondary Traumatic Stress (STS), each with ten statements from the total 30 statement scale. CS is generally defined as the pleasure one derives from being able to do one’s work well (protective factor). BO refers to feelings of hopelessness and difficulties in dealing with work due to being fatigued by stress and the toll helping-related work takes. STS is analogous with Post Traumatic Stress (PTS) with one main difference. STS has its etiology in helping others or patients who have experienced traumatic events, whereas PTS is about the individual who has experienced a traumatic event.

Higher scores on the ProQoL indicate higher CS, BO, or STS. For interpretation purposes, we categorized respondents as Low (22 or less), Moderate (23–41), and High (42 or more) for each subscale with cut scores derived from validation studies with multiple healthcare or helping professions [20]. Due to the small number of responses, we combined High (n = 5) with Moderate STS and combined Low (n = 6) with Moderate CS. No respondents reported high BO, so this variable is presented as Low and Moderate.

We included two Covid–specific measures, the Coronavirus Anxiety Scale (CAS) [21], and the Obsession with Coronavirus Scale (OCS) [22]. The CAS is a five-item scale used to screen for dysfunctional anxiety associated with coronavirus and contains questions like “I felt dizzy, lightheaded, or faint, when I read or listened to the news about the coronavirus”. The Obsession with Coronavirus Scale is a four-item scale that screens for persistent and disturbed thinking about COVID-19. It contains questions like “I could not stop thinking about the coronavirus”. Both the CAS and the OCS are rated as “0” meaning “not at all” to “4” meaning Nearly every day over the last 2 weeks” with higher scores meaning more severe anxiety or obsessive thoughts respectively. The CAS has a cut score of “9” whereas “7” or above on the OCS indicates more severe concerns. We used the recommended cut scores to categorize study participants into dichotomous groups of probable or not probable for the purposes of this study.

Descriptive statistics were calculated for demographic, occupational, life circumstances, and health characteristics. We ran univariate analyses to assess the association between the four outcome variables 1) PTSD, 2) Burnout 3) Secondary Traumatic Stress and 4) Compassion Satisfaction with the independent variables. Statistical differences were examined by Chi-square test for categorical variables, and the mean and standard deviation was reported for continuous variables. Multivariable logistic regression analyses were used to examine the demographic, health, life circumstance, and occupational factors associated with each outcome during the study period. Each model included the same set of independent variables accounting for demographic characteristics (sex, age group, race, ethnicity), occupational characteristics (occupational group, years of experience), prior behavioral health or emotional health concerns, and COVID-19 variables (caring for a patient who died from COVID-19, having probable COVID-19 obsession). We used SAS statistical software version 9.4 for all analyses and set the significance level at α = 0.05.

## Results

The majority of the survey population identified as female (71%), were from racial minority groups (78%), and over a third were Hispanic/Latino/x ethnicity. There was a similar proportion (24%) of respondents in each age group, except the 5% of respondents who were 65 years of age and older. Non-clinical personnel represented 33%, clinical staff represented 51% (e.g. nursing staff, physicians, and nurse practitioners), and allied health professionals (e.g. laboratory, social work) represented 16% of the survey population. Seventy percent had a bachelor or graduate degree and 30% had 20 years or more job experience (Table 1).

**Table 1:** Demographic, occupational, life circumstance and health characteristic of Lincoln Hospital survey respondents (n = 889).

Variables	N (%)
**Sex** ^a^ ^,^^	
Male	239 (27)
Female	624 (71)
Other^b^	17 (2)
**Age** ^a^ ^,†,×^	
<35 years	212 (24)
35–44 years	206 (23)
45–54 years	221 (25)
55–64 years	203 (23)
65+ years	45 (5)
**Race** ^a^ ^,†,×^	
Asian	189 (22)
Black	288 (34)
Multiple/Other	182 (22)
White	190 (22)
**Ethnicity**	
Latinx	299 (34)
Non-Latinx	518 (58)
Other^c^	72 (8)
**Education level** ^a^	
High school degree or lower	55 (6)
Some college but no degree	94 (11)
Associate degree	115 (13)
Bachelor degree	257 (29)
Graduate degree	364 (41)
**Primary role in the hospital**^d^^,^,^*^,†,×^	
Administration and Support	293 (33)
Allied Healthcare Worker	139 (16)
Medical Provider	188 (21)
Nursing Staff	267 (30)
**Years of experience in the profession**^a^^,^,^*^,†,×^	
< 5 years	248 (28)
6–10 years	139 (16)
11–15 years	138 (16)
16–20 years	95 (10)
20+ years	264 (30)
**Clinical care to COVID-19 patients** ^a^ ^,†^	
No	350 (40)
Yes	532 (60)
**Clinical care to COVID-19 patients who died**^a^^,^*^,†^	
No	470 (53)
Yes	415 (47)
**Tested positive for COVID-19?** ^a^	
No	693 (78)
Yes	193 (22)
**Family member or close friend tested positive**^a^^,^,^*	
No	380 (43)
Yes	508 (57)
**Prior behavioral/emotional health concern**^a^^,^,^*^,†,×^	
No	800 (91)
Yes	81 (9)
**Living at home with a high risk person** ^†^	
No	300 (34)
Yes	589 (66)
**Role changed during outbreak** ^†^	
No	477 (54)
Yes	405 (46)
**Work where COVID-19 patients being tested/treated** ^a^	
No	63 (7)
Yes	823 (93)
**Coronavirus Anxiety**^^,^*^,†^	
No Anxiety	832 (94)
Probable Anxiety	57 (6)
Mean (Standard Deviation [STD])	2.70 ± 3.69
**Coronavirus Obsession**^^,^*^,†^	
No Obsession	706 (79)
Probable Obsession	183 (21)
Mean ± STD	3.78 ± 3.86
Post-Traumatic Stress Disorder (PTSD)*^,†,×^	
No PTSD	770 (87)
Probable PTSD	117 (13)
Mean ± STD	1.29 ± 1.587
**Secondary Traumatic Stress (STS)** ^a^ ^,^ ^e^ ^,†,×^	
Low	485 (55)
Moderate	403 (45)
Mean ± STD	22.29 ± 7.00
**Burnout**^a^^,^*^,^	
Low	628 (71)
Moderate	260 (29)
Mean ± STD	19.75 ± 5.67
**Compassion Satisfaction**^a^^,^^f^^,^,^*^,†,×^	
High	465 (52)
Moderate	423 (48)
Mean (Standard Deviation)	41.09 ± 6.55

^a^Variable has missing information.

^b^Includes don’t know, choose not to disclose, and missing.

^c^Includes prefer not to answer and those who identify as transgender.

^d^Administrative and support (e.g. security, administration and other support staff), nursing staff (e.g. RNs and other patient care staff), medical providers (e.g. physicians, residents, nurse practitioners, physician assistants), allied health professionals (e.g. behavioral health, laboratory).

^e^Recoded variable to include “high” STS in the moderate category due to the small number (n = 5) of responses in the high category.

^f^Recoded variable to include low compassion satisfaction in the moderate category due to small number (n = 6) of responses in the low category. *p < 0*.*05* at the univariate level for ^^^PTSD,*STS,^†^Burnout,^×^compassion satisfaction.

Occupationally, almost half (46%) reported that their work-role changed as a result of the pandemic response and 47% indicated they provided clinical care to COVID-19 patients who died. Almost a quarter (22%) reported testing positive for COVID-19 and 57% said a family member or friend tested positive. For the main outcomes, 13% (n = 117) of respondents screened positive for Probable PTSD, 45% (n = 403) reported moderate or high STS, 25% (n = 206) reported moderate burnout and 54% reported high compassion satisfaction. For other adverse mental health outcomes, 91% did not have a behavioral or emotional health concern before the pandemic and 21% endorsed obsessive thoughts about COVID-19 (Table 1).

In the adjusted logistic regression analyses, females had significantly higher odds of having probable PTSD compared to males. Coronavirus obsession, moderate/high STS, moderate burnout, and those with a prior behavior or emotional health concern were also independently associated with increased odds of having probable PTSD (Table 2).

**Table 2:** Adjusted multivariable logistic regression models for post-traumatic stress disorder (PTSD), secondary traumatic stress (STS), burnout and compassion satisfaction among Lincoln Hospital survey respondents.

Variable	Probable PTSD^a^ *aOR* (95% CI)	Moderate/High STS^b^ *aOR* (95% CI)	Moderate BO^c^ *aOR* (95% CI)	High CS^d^ *aOR* (95% CI)
**Sex** ^e^				
Male	ref	ref	ref	Ref
Female	**2.14 (1.10–4.15)**	1.20 (0.81–1.77)	1.00 (0.62–1.61)	1.02 (0.69–1.48)
Other^f^	4.19 (0.86–20.32)	1.03 (0.28–3.75)	0.57 (0.12–2.78)	0.86 (0.24–2.98)
**Age** ^e^				
<35 years	ref	ref	ref	ref
35–44 years	1.22 (0.59–2.48)	0.91 (0.56–1.47)	0.86 (0.50–1.48)	1.30 (0.81–2.10)
45–54 years	2.13 (1.02–4.44)	1.04 (0.63–1.71)	**0.38 (0.21**–**0.69)**	1.51 (0.93–2.46)
55–64 years	0.93 (0.41–2.10)	1.16 (0.70–1.94)	0.55 (0.30–1.01)	1.63 (0.98–2.70)
65+ years	0.33 (0.04–3.04)	1.03 (0.63–1.71)	0.33 (0.10–1.04)	2.78 (1.22–6.33)
**Race** ^e^				
White	ref	ref	ref	ref
Asian	1.35 (0.60–3.06)	1.74 (1.03–2.94)	0.67 (0.36–1.29)	0.99 (0.59–1.66)
Black	2.05 (0.97–4.31)	1.30 (0.79–2.14)	0.47 (0.26–0.83)	1.41 (0.87–2.27)
Multiple/Other	1.06 (0.45–2.50)	1.40 (0.82–2.38)	0.63 (0.33–1.19)	1.50 (0.90–2.52)
**COVID patient died**				
No	ref	ref	ref	ref
Yes	0.91 (0.50–1.65)	1.38 (0.92–2.09)	1.49 (0.89–2.48)	**1.63 (1.09–2.44)**
**Prior BH/EH concern** ^e^				
No	ref	ref	ref	ref
Yes	**2.01 (1.00–4.04)**	1.16 (0.62–2.16)	**2.48 (1.28–4.81)**	1.15 (0.62–2.13)
**Coronavirus Obsession**				
No Coronavirus Obsession	ref	ref	ref	ref
Probable Coronavirus Obsession	**3.93 (2.35–6.56)**	**3.84 (2.44–6.03)**	1.56 (0.93–2.60)	1.44 (0.91–2.28)
**PTSD**				
No PTSD	N/A	ref	ref	ref
Probable PTSD	N/A	**6.72 (3.27–13.81)**	**3.88 (2.11–7.11)**	0.87 (0.49–1.57)
**STS** ^e^				
Low	ref	N/A	ref	ref
Moderate or High	**6.58 (3.19–13.57)**	N/A	**4.12 (2.68–6.32)**	**0.67 (0.47–0.96)**
**Burnout** ^e^				
Low	ref	ref	N/A	**ref**
Moderate	**4.03 (2.15–7.56)**	**3.92 (2.57–5.98)**	N/A	**0.11 (0.07–0.17)**
**Compassion Satisfaction** ^e^				
Low or Moderate	1.17 (0.63–2.17)	**1.48 (1.03–2.13)**	**9.70 (6.18–15.22)**	N/A
High	ref	**ref**	ref	N/A

Note: PTSD: Post-Traumatic Stress Disorder; STS: Secondary Traumatic Stress; BO: Burnout; CS: Compassion Satisfaction; BH: Behavioral health; EH: Emotional Health. Each model included the same set of independent variables accounting for demographic characteristics (sex, age group, race, ethnicity), occupational characteristics (occupational group, years of experience), prior behavioral health or emotional health concerns, and COVID-19 variables (caring for a patient who died from COVID-19, having probable COVID-19 obsession). Bolded ORs and CI’ represent significant variables. Ethnicity and occupational group were not significant in any of the models and are not show.

^a^No PTSD v. Probable PTSD.

^b^Moderate/High STS v. Low STS.

^c^Moderate BO v. Low STS.

^d^High CS v. Moderate CS.

^e^Variable has missing information.

^f^Other includes prefer not to answer and those who identify as transgender.

Results from the STS model showed that HCWs with coronavirus obsession, probable PTSD, moderate burnout, and high compassion satisfaction all had significantly higher odds of reporting moderate/high STS (Table 2). HCWs 45–54 years of age were significantly less likely to have moderate BO.

Respondents with a prior behavioral or emotional health concern, probable PTSD, moderate/high STS, and low/moderate compassion satisfaction had significantly higher odds of reporting moderate burnout (Table 2).

Finally, three variables were associated with HCWs having significantly higher odds of reporting high compassion satisfaction: caring for a patient that died from COVID-19, low burnout, and low STS (Table 2). Of note, occupation group, race, nor ethnicity were significant in any of the four models.

## Discussion

Current evidence and theory suggest that most individuals will not have any significantly disturbing post-traumatic symptoms, and of those that do, those symptoms tend to dissipate without intervention [23]. COVID-19 is an outlier in that it pervasively intrudes on work, personal, and social life and is a chronic stressor in that the death toll and stress on everyday life worsened as 2020 progressed. As of this writing, the United States is now one year into living in the COVID-19 era and the toll on lives lost, economic suffering, and social isolation has left a psychological mark on not just healthcare workers, but a generation.

While previous studies have focused on understanding the adverse mental health problems of frontline healthcare workers during COVID-19, this study included other essential hospital staff that may have been affected by the pandemic. Additionally, 78% of the population were minorities. This study highlights how important prior behavioral or emotional health, age differences, and occupational factors are to the larger research context of healthcare worker mental health. Understanding the full picture of risk and protective factors will be critical to crafting a response to address and support the needs of the healthcare community. This study supports previous literature finding higher than expected rates of adverse mental health problems among healthcare workers during COVID-19 [24]. In particular, our finding of negative mental health outcomes among women and younger healthcare workers is consistent with earlier reports [2, 3, 6, 10, 13]. We also found protective factors such as low burnout and higher compassion satisfaction. We observed that being a frontline worker who cared for a patient that died, was uniquely associated with high compassion satisfaction, a topic unexplored in previous studies.

Placing these findings in the broader context of HCW mental health is important. First, there is wide variability in the experience of trauma-inducing events among healthcare workers [19]. Second, burnout is perhaps the most commonly referenced mental health concern for healthcare workers. In a pre-COVID-19 national sample of over 30,000 US physicians, approximately 44% reported symptoms of burnout, which was higher than the general population estimate [25]. Reports also routinely flag depression, suicidality, and general stress, especially in physician and nursing healthcare workers [26]. Alternatively, higher levels of education, higher incomes, and better overall socioeconomic factors make HCWs, as a group, more resilient to stress as compared to the general American population.

Our study’s findings have implications for hospital preparedness for operating during emergencies of this scale. Healthcare leaders and managers should include workforce sustainment strategies in their overall crisis response plans [27, 28]. First, hospitals must account for the groups of HCWs that may be more vulnerable to the high-stress environment and are likely to be at increased risk for poor mental health outcomes. Planning for additional supportive services could help offset some of these concerns. For example, prior findings highlighting women at disproportionate risk may be tied to other stressors such as childcare burdens exacerbated by COVID-19 (schools were closed) [1].

The largest proportion of medical providers (15%) were <44 years of age. The observation that younger staff were at increased risk for burnout might indicate that medical residents need additional support from formal mentors or peer support structures. Almost 10% of respondents indicated a pre-existing behavioral health concern, and our data showed that this was a unique risk factor for moderate burnout. Hospitals could alert staff to consider whether they fall into this category, make additional supports available, and urge staff members to avail themselves of support. This is of particular importance given the consistent observation that clinical staff are less likely to seek mental health support in general [26] and may put their own well-being to the side in crisis situations.

## Limitations

This study has limitations to consider. First, using a single-location sample limits generalizability. Specifically, we sampled workers at one hospital in New York City. NYC is unfortunately experienced with mass trauma, which could have muted or amplified some of the trauma and resilience findings. As noted earlier, we contend that this type of study is most appropriate for local understanding and intervention and should be applied cautiously to other locations. Future research with generalizability as a goal might use a multi-site/location approach to help mitigate any location-based bias in the sample.

Second, the self-report measures in our study carry with them limitations common to self-report assessment, such as the accuracy of the respondent’s ability to assess and report their attitudes, responses, and symptoms, in addition to any uncontrolled bias in responses or sampling procedures. We used standardized and validated measures to mitigate these threats, but they are not perfect. Our goal was to characterize the sample, and as such, we prioritized sensitivity over specificity when selecting measures. Future studies might consider using more robust measures that prioritize specificity or diagnosing in the case of post-traumatic stress.

Finally, while we made every effort to achieve maximum participation, the 24% response rate may not be entirely reflective of the hospital population. Workers who do not routinely use computers, as an example, may be underrepresented. Additionally, HCWs out due to shift work, illness, or personal concerns may have contributed to the relatively low participation rate. While we can’t be certain, workers who did not or could not participate may have also had differing views from those who did participate in this study and, therefore, might have altered the results. Future studies should aim for a better participation rate, especially in defined-location studies. This step will increase the likelihood that the findings represent the attitudes of the population.

## Supporting information

S1 Data (XLSX)
